# Characterization of Binding and Inhibitory Properties of TAK-063, a Novel Phosphodiesterase 10A Inhibitor

**DOI:** 10.1371/journal.pone.0122197

**Published:** 2015-03-27

**Authors:** Akina Harada, Kazunori Suzuki, Naomi Kamiguchi, Maki Miyamoto, Kimio Tohyama, Kosuke Nakashima, Takahiko Taniguchi, Haruhide Kimura

**Affiliations:** 1 Central Nervous System Drug Discovery Unit, Pharmaceutical Research Division, Takeda Pharmaceutical Company Limited, Fujisawa, Japan; 2 Drug Metabolism & Pharmacokinetics Research Laboratories, Pharmaceutical Research Division, Takeda Pharmaceutical Company Limited, Fujisawa, Japan; 3 Discovery Research Laboratories, Pharmaceutical Research Division, Takeda Pharmaceutical Company Limited, Fujisawa, Japan; IDIBAPS, SPAIN

## Abstract

Phosphodiesterase 10A (PDE10A) inhibition is a novel and promising approach for the treatment of central nervous system disorders such as schizophrenia and Huntington’s disease. A novel PDE10A inhibitor, TAK-063 [1-[2-fluoro-4-(1*H*-pyrazol-1-yl)phenyl]-5-methoxy-3-(1-phenyl-1*H*-pyrazol-5-yl)-pyridazin-4(1*H*)-one] has shown high inhibitory activity and selectivity for human recombinant PDE10A2 *in vitro*; the half-maximal inhibitory concentration was 0.30 nM, and selectivity over other phosphodiesterases (PDEs) was more than 15000-fold. TAK-063 at 10 µM did not show more than 50% inhibition or stimulation of 91 enzymes or receptors except for PDEs. *In vitro* autoradiography (ARG) studies using rat brain sections revealed that [^3^H]TAK-063 selectively accumulated in the caudate putamen (CPu), nucleus accumbens (NAc), globus pallidus, substantia nigra, and striatonigral projection, where PDE10A is highly expressed. This [^3^H]TAK-063 accumulation was almost entirely blocked by an excess amount of MP-10, a PDE10A selective inhibitor, and the accumulation was not observed in brain slices of *Pde10a*-knockout mice. In rat brain sections, [^3^H]TAK-063 bound to a single high-affinity site with mean ± SEM dissociation constants of 7.2 ± 1.2 and 2.6 ± 0.5 nM for the CPu and NAc shell, respectively. Orally administered [^14^C]TAK-063 selectively accumulated in PDE10A expressing brain regions in an *in vivo* ARG study in rats. Striatal PDE10A occupancy by TAK-063 *in vivo* was measured using T-773 as a tracer and a dose of 0.88 mg/kg (p.o.) was calculated to produce 50% occupancy in rats. Translational studies with TAK-063 and other PDE10A inhibitors such as those presented here will help us better understand the pharmacological profile of this class of potential central nervous system drugs.

## Introduction

Intracellular cAMP and cGMP play important roles as second messenger molecules controlling multiple cellular processes. Phosphodiesterases (PDEs) are enzymes that regulate cellular levels of these cyclic nucleotides by regulating their degradation rates [1]. There are 11 different families of PDEs comprising 21 different gene products, and each PDE superfamily enzyme shows a distinct distribution pattern and has important functions [1]. PDE10A is a dual-substrate PDE that hydrolyzes both cAMP and cGMP [2,3], and it is highly enriched in the central nervous system (CNS) of many mammalian species including humans [4,5]. In the mammalian brain, PDE10A mRNA and protein are selectively expressed in striatal medium spiny neurons (MSNs) [1,2,4,5].

The striatal outputs mediated by MSNs are divided into two pathways: the dopamine D_2_ receptor expressing indirect pathway and the D_1_ receptor expressing direct pathway [6,7]. Activation of the indirect pathway by D_2_ receptor antagonism is thought to be the principal mechanism of action of most antipsychotic drugs [8]; however, excessive activation of the indirect pathway by D_2_ receptor antagonists is known to cause extrapyramidal symptoms (EPS) [9]. Activation of the direct pathway is expected to counteract excessive activation of the indirect pathway and reduce these side effects [10]. In line with this idea, PDE10A inhibitors have shown lower risks of EPS through the activation of both direct and indirect pathways in pre-clinical studies [11]. In addition to EPS, some of the current antipsychotics cause hyperprolactinemia owing to their D_2_ receptor antagonism in the pituitary gland [12]. PDE10A inhibitors can avoid hyperprolactinemia as PDE10A expression is low in the pituitary gland. Furthermore, PDE10A inhibitor can modulate cognitive functions via activation of corticostriatal circuit [7,13]. Accordingly, PDE10A inhibition can be a novel therapeutic approach for the treatment of schizophrenia with lower risks of these side effects [10,14,15]. Moreover, several pre-clinical studies have shown that PDE10A inhibitors can protect striatal MSNs against neurodegeneration in Huntington’s disease (HD) models through the improvement of cAMP signaling [16–18]. Thus, we decided to develop a PDE10A inhibitor as a therapeutic drug for the abovementioned CNS disorders.

Each PDE family has essential functions; thus, PDE10A selectivity is critical for avoiding off-target effects associated with inhibition of other PDEs. For instance, PDE4 inhibition in the brainstem is thought to cause emesis [19], and PDE6 inhibition in the mammalian retina can cause disturbance in visual function [20]. PDE10A selectivity is also crucial in understanding the pharmacological profile of PDE10A inhibitors because small molecules targeting distinct PDEs show overlapping pharmacological effects. For example, both the PDE4 inhibitor rolipram and the PDE5 inhibitor zaprinast enhance memory functions in novel object recognition tests (NORT) using mice [21]. Both the PDE2 inhibitor BAY 60–7550 and the PDE10A inhibitor PQ-10 attenuate scopolamine- and MK-801-induced memory deficits in NORT using rats [22]. In addition, The PDE4 inhibitor RO 20–1724, the PDE5 inhibitor sildenafil, and the PDE10 inhibitor TP-10 ameliorate motor dysfunction in rodent HD models [17,23]. Thus, careful validation of PDE10A selectivity under physiological conditions is crucial for the precise profiling of PDE10A inhibitors.

TAK-063 [1-[2-fluoro-4-(1*H*-pyrazol-1-yl)phenyl]-5-methoxy-3-(1-phenyl-1*H*-pyrazol-5-yl)-pyridazin-4(1*H*)-one] was discovered as a novel PDE10A inhibitor by optimization using a structure-based drug design strategy [24]. In the present study, we investigated the PDE10A selectivity of TAK-063 using multiple methods, including *in vitro* and *in vivo* autoradiography (ARG) in rodents. Finally, we assessed PDE10A occupancy by TAK-063 in rats using T-773 as a tracer; [^11^C]T-773 is our original PET radioligand for PDE10A [25]. Translational studies with TAK-063 based on the information presented here will help us to understand the pharmacological profile of PDE10A inhibitors as potential CNS drugs.

## Materials and Methods

### Ethics Statement

The care and use of the animals and the experimental protocols used in this research were approved by the Experimental Animal Care and Use Committee of Takeda Pharmaceutical Company Limited.

### Animals

Seven-week-old male Sprague–Dawley (SD) rats were purchased from Charles River Laboratories Japan, Inc. (Yokohama, Japan). After acclimation for 1 week in our laboratory, the 8-week-old rats were used for experiments. *Pde10a* wild-type (WT) and homozygous knockout (KO) mice (129/SvEv-C57BL/6) were purchased from Taconic Farms, Inc. (Hudson, NY), and used for experiments after at least 1 week of acclimation. The animals were housed in a light-controlled room (12-h light/dark cycle with lights on from 7:00 AM). Food and water were provided ad libitum.

### Radioligands and Chemicals

TAK-063, MP-10 succinate, and T-773 were synthesized by Takeda Pharmaceutical Company Limited. MP-10 has been reported to be a potent and selective PDE10A inhibitor developed by Pfizer Inc. (New York City, NY) [11,26,27]. [^3^H]TAK-063 (37.0 MBq/mL in ethanol) was synthesized by Sekisui Medical Co., Ltd. (Tokyo, Japan). The specific radioactivity and radiochemical purity were 665 GBq/mmol and 98.1%, respectively. [^14^C]TAK-063 was synthesized by Nemoto Science Co., Ltd. (Tokyo, Japan). The specific radioactivity and radiochemical purity were 3.08 GBq/mmol and 99.2%, respectively. [^3^H]T-773 (37.0 MBq/mL in ethanol) was synthesized by Quotient Bioresearch (Radiochemicals) Limited (Cambridgeshire, UK). The specific radioactivity and the radiochemical purity were 555 GBq/mmol and 99.9%, respectively.

### Preparation of Tissue Slices

Male SD rats and male *Pde10a* WT and KO mice were euthanized by decapitation. The brains were rapidly removed, slowly frozen in an isopentane-dry ice bath, and then stored in a deep freezer. Sagittal or coronal 20-μm-thick sections were cut in a cryostat (Leica Microsystems, Wetzlar, Germany) and thaw-mounted onto glass slides. For the rat, coronal brain sections were collected from the region between 1.7 to 0.2 mm anterior to bregma, and sagittal sections were collected from the region 1.9–3.4 mm lateral to the midline [28]. For the mouse, coronal brain sections were collected from the region between 1.5 to 0.7 mm anterior to bregma [29].

### *In Vitro* ARG Using Rat and Mouse Brain Sections

Sagittal brain sections prepared from a rat brain or coronal brain sections prepared from *Pde10a* WT and KO mouse brains (n = 5 per genotype) were warmed to room temperature. The sections were pre-incubated in buffer (50 mM Tris-HCl pH 7.5, 1.7 mM EDTA, 6 mM MgCl_2_, 120 mM NaCl and 0.1% BSA) twice for 5 min at room temperature. The sections were then incubated in binding buffer (pre-incubation buffer containing 0.03% Triton X-100) with [^3^H]TAK-063 (8 nM for rats and 16 nM for mice) or [^3^H]T-773 (20 nM for rats) for 60 min at room temperature. Blocking of specific binding in adjacent sections was conducted by the addition of an excess amount of PDE10A-selective inhibitors to the radioligand-containing buffer (final concentration of 1 μM). The sections were washed twice for 5 min ([^3^H]TAK-063) or for 1 min ([^3^H]T-773) at 4°C in pre-incubation buffer, and then rapidly rinsed in ice-cold distilled water. The sections were dried under a stream of cool air, and were exposed to BAS IP TR 2040E imaging plates (GE Healthcare UK Ltd.) for 5–7 days. The imaging plates were analyzed using an image analyzer FLA-7000 (Fujifilm, Tokyo, Japan) and image analyzing software ImageGauge 4.0 (Fujifilm). In the ARG study using [^3^H]TAK-063, regions of interest (ROIs) were placed at the frontal cortex (Fcx), caudate putamen (CPu), nucleus accumbens (NAc), thalamus (Thal), brainstem (Bs), hippocampus (Hipp), and cerebellum (Cb). Radioactivity in each ROI was analyzed and represented as photostimulated luminescence (PSL) values. The background was subtracted from the PSL values of each ROI, and the PSL values in each brain region were then averaged for each group. In the ARG study in mouse brain sections, the PSL values in the presence and absence of an excess amount of MP-10 were represented as total binding and non-specific binding (NSB), respectively. The rat brain sections adjacent to those used for ARG were stained with hematoxylin and eosin (HE) for anatomical identification.

### Saturation Binding Assay with [^3^H]TAK-063 Using Rat Brain Sections

Coronal brain sections prepared from 4 rat brains were warmed to room temperature, and were pre-incubated twice in buffer for 5 min at room temperature. The sections were then incubated with 0.25, 0.5, 1, 2, 4, 8, 16, or 32 nM [^3^H]TAK-063 in binding buffer for 60 min at room temperature. NSB was determined in the presence of 1 μM MP-10. The sections were rinsed, dried using the same procedure described above, and then exposed to an imaging plate for 6 days. Autoradiograms were read using FLA-7000 and analyzed using ImageGauge 4.0. ROIs were placed at the CPu and NAc shell of both hemispheres in each section and radioactivity in the ROIs was represented as PSL values. The background PSL value was subtracted from the PSL values of each ROI, and the PSL values of the left and right hemispheres were averaged for each section. The PSL values in the absence and presence of an excess amount of MP-10 were represented as total binding and NSB, respectively. The saturation binding curves were fit by nonlinear regression using GraphPad Prism 5.01 (GraphPad Software, Inc., La Jolla, CA), and the dissociation constant (K_d_ value) was calculated using the same software.

### *In Vivo* ARG with [^14^C]TAK-063 in Rats

[^14^C]TAK-063 was suspended in 0.5% (w/v) methylcellulose in distilled water and orally administrated to male SD rats (n = 2) at 1.5 mg/kg (10.1 MBq/9.48 mL/kg). At 6 h after administration, the rats were euthanized by inhalation of chloroform under anesthesia with isoflurane, and were preliminarily frozen in a bath of dry ice/hexane. The decapitated head was embedded in 2% (w/v) sodium carboxymethyl cellulose in distilled water. Using a cryostat, 40-μm-thick sagittal sections were collected from the right hemispheres of the heads, and then 40-μm-thick coronal sections were collected from the left hemispheres. These sections were freeze-dried in a cryostat at approximately—20°C for 1 day. The sections were then covered with a sample-protecting film (Nakagawa Mfg. Co., Ltd., Warabi, Japan) and were exposed to an imaging plate BAS-MS2040 or BAS-III2040 (GE Healthcare UK Ltd.) for 48 h. After the exposure, the imaging plate was analyzed with an image analyzer FLA-7000 (Fujifilm).

### *In vivo* Occupancy Study of TAK-063 in Rats

TAK-063 was suspended in 0.5% (w/v) methylcellulose in distilled water, and T-773 was dissolved in *N*,*N*-dimethylacetamide and 1,3-butanediol (1:1). TAK-063 (0, 0.03, 0.1, 0.3, 1, 3, and 10 mg/kg) was orally administered to male SD rats (n = 2–3 in each group), and 0.02 mg/kg of T-773 was administered by bolus intravenous injection via the lateral tail vein 90 min after TAK-063 administration. The rats were anesthetized by inhalation of 4% isoflurane and were euthanized by cardiac perfusion with heparinized saline 30 min after T-773 injection, and the whole brains were isolated. The striatum (Str) and cerebellum (Cb) were dissected from the brains, and were stored at −30°C until use. The frozen samples were homogenized in saline at 4 mL/g tissue, and the concentration of T-773 was measured by mass spectrometry (MS) in each homogenate. Specific T-773 binding (B_SP_) in Str was represented as the difference between the T-773 concentration in Str and that in Cb. PDE10A occupancy was calculated using the following equation: Occupancy (%) = (B_SP,base_ − B_SP,drug_)/B_SP,base_ × 100, where B_SP,base_ and B_SP,drug_ are the concentrations at baseline (vehicle-treatment) and at drug-treatment, respectively. The saturation curve of occupancy was fit by nonlinear regression using GraphPad Prism 5.02.

## Results

### *In Vitro* PDE10A Selectivity of TAK-063

TAK-063 was identified as a novel PDE10A inhibitor. We have reported the PDE10A2 inhibitory activity of TAK-063 and its selectivity over other PDE family enzymes by *in vitro* enzyme inhibition assays using various human recombinant PDE family proteins [24]. The half-maximal inhibitory concentration (IC_50_) value of TAK-063 for PDE10A2 was 0.30 nM, and the minimum IC_50_ value among the other 10 PDE families was 5500 nM for PDE4D2. Thus, the PDE10A2 selectivity of TAK-063 over other PDE family enzymes was more than 15000-fold. *In vitro* PDE10A2 selectivity of TAK-063 was further assessed by measuring its inhibitory or stimulatory activities against enzymes (Table 1) and receptors (Table 2) at Ricerca Biosciences (Concord, OH). More than 50% inhibition or stimulation by 10 μM of TAK-063 was considered as a significant response. TAK-063 did not induce a significant response in 91 target molecules, except for PDEs. These results indicate that TAK-063 is a potent and selective inhibitor of human PDE10A *in vitro*.

**Table 1:** Percent inhibition of enzymes by TAK-063 at 10 μM.

Enzyme	% inhibition
Acetylcholinesterase	3
ATPase, Ca^2+^, Skeletal Muscle	6
ATPase, Na^+^/K^+^, Heart	8
Carbonic anhydrase II	6
Cyclooxygenase-1 (COX-1)	26
Cyclooxygenase-2 (COX-2)	6
EGF receptor tyrosine kinase	5
HMG-CoA reductase	11
5-Lipoxygenase (5-LO)	3
Monoamine oxidase A (MAO-A)	16
Monoamine oxidase B (MAO-B)	10
Nitric oxide synthase, inducible (iNOS)	1
Nitric oxide synthase, neuronal (nNOS)	12
Peptidase, factor Xa	1
Matrix metalloproteinase-1 (MMP-1)	9
Matrix metalloproteinase-7 (MMP-7)	7
Matrix metalloproteinase-13 (MMP-13)	11
Phosphodiesterase PDE3	23
Phosphodiesterase PDE4	50
Phosphodiesterase PDE5	44
Phosphodiesterase PDE6	20
Phosphodiesterase PDE10A1	101
Protein kinase A (PKA), nonselective	−3
Protein kinase C (PKC), nonselective	7
Steroid 5α-reductase	16
Xanthine oxidase	−3

EGF, epidermal growth factor; HMG CoA, 3-hydroxy-3-methyl-glutaryl coenzyme A. Negative value of percent inhibition indicates activation of enzyme activity.

**Table 2:** Percent inhibition of receptors by TAK-063 at 10 μM.

Receptor	% inhibition
Adenosine A_1_	11
Adenosine A_2A_	−4
Adenosine A_2B_	0
Adrenergic α_1_, non-selective	14
Adrenergic α_2_, non-selective	−2
Adrenergic β_1_	6
Adrenergic β_2_	6
Adrenergic β_3_	1
Androgen (testosterone)	6
Angiotensin AT_1_	13
Angiotensin AT_2_	−4
Bradykinin B_1_	11
Bradykinin B_2_	−2
Calcium channel L-type, benzothiazepine	3
Calcium channel L-type, dihydropyridine	0
Calcium channel L-type, phenylalkylamine	4
Calcium channel N-type	−3
Cannabinoid CB_1_	3
Cholecystokinin CCK_1_ (CCK_A_)	5
Cholecystokinin CCK_2_ (CCK_B_)	1
Dopamine D_1_	8
Dopamine D_2L_	−7
Dopamine D_3_	−1
Dopamine D_4.2_	7
Dopamine transporter (DAT)	11
Estrogen ERα	3
GABA_A_, chloride channel	12
GABA_A_, flunitrazepam, central	−3
GABA_A_, muscimol, central	0
GABA_A_, non-selective	7
GABA_B1A_	−18
GABA_B1B_	−13
GABA transporter	11
Glucocorticoid	−5
Glutamate, AMPA	−6
Glutamate, kainate	5
Glutamate, NMDA	24
Glutamate, NMDA, glycine	11
Glutamate, NMDA, phencyclidine	0
Glycine, strychninee	−1
Growth hormone secretagogue (ghrelin)	1
Histamine H_1_	−8
Histamine H_2_	−5
Imidazoline I_2_, central	7
Insulin	−8
Muscarinic M_1_	4
Muscarinic M_2_	−10
Muscarinic M_3_	−1
Nicotinic acetylcholine	1
Norepinephrine transporter (NET)	15
Opiate δ (OP1, DOP)	16
Opiate κ (OP2, KOP)	5
Opiate μ (OP3, MOP)	−1
Potassium channel (K_ATP_)	12
Potassium channel (SK_CA_)	5
Progesterone PR-B	0
Prostanoid / thromboxane A_2_ (TP)	13
Serotonin 5-HT_1_, non-selective	15
Serotonin 5-HT_2_, non-selective	23
Serotonin 5-HT_2B_	14
Serotonin 5-HT_3_	−3
Serotonin 5-HT_4_	−1
Serotonin transporter (SERT)	−5
Sigma, non-selective	−9
Sodium channel, Site 2	15
Tachykinin NK_1_	−2
Tachykinin NK_2_	0
Tachykinin NK_3_	−11
Vasopressin V_1A_	4
Vasopressin V_2_	−2

AMPA, α-amino-3-hydroxy-5-methyl-4-isoxazolepropionic acid; NMDA, N-methyl-D-aspartic acid. Negative value of percent inhibition indicates stimulation of receptor activity.

### *In Vitro* ARG with [^3^H]TAK-063 in Rat Brain Sections

**Fig 1:**
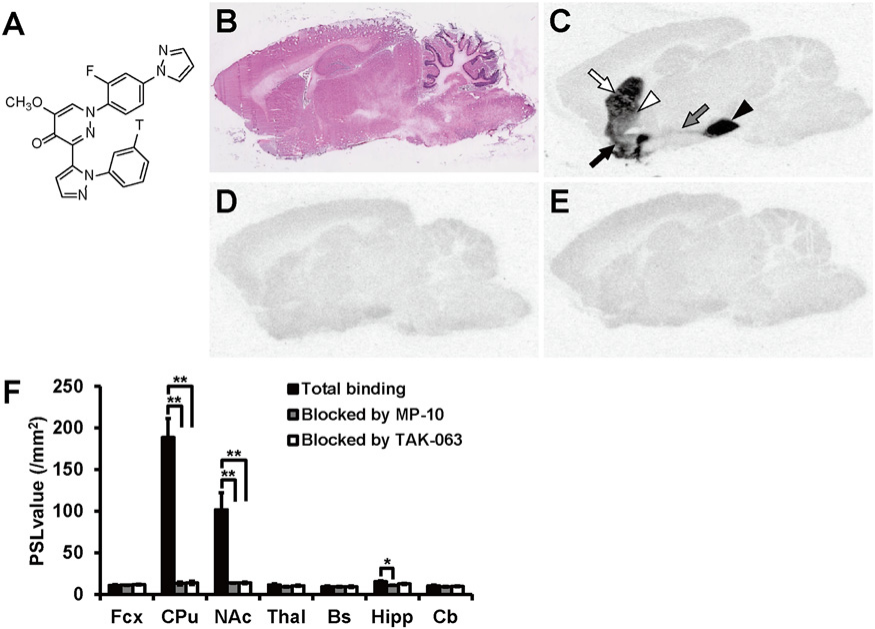
*In vitro* autoradiography (ARG) using [^3^H]TAK-063 in sagittal rat brain sections. The chemical structure of [^3^H]TAK-063 (A). Sections adjacent to those used for *in vitro* ARG of [^3^H]TAK-063, were stained with hematoxylin and eosin (B). The autoradiogram shows the high accumulation of [^3^H]TAK-063 in the caudate putamen (CPu; white arrow), nucleus accumbens (NAc; black arrow), globus pallidus (GP; white arrow head), substantia nigra (SN; black arrow head), and striatonigral projection (gray arrow; C). *In vitro* ARGs in the presence of an excess amount of MP-10 (D) or TAK-063 (E) were performed with adjacent sections. Radioactivity levels in several brain regions were represented as photostimulated luminescence (PSL) values in the presence or absence of an excess amount of MP-10 or TAK-063 (F). Statistical analyses were performed using Dunnett's test (*P ≤ 0.05, **P ≤ 0.01 vs total binding, n = 3). Fcx, frontal cortex; Thal, thalamus; Bs, brainstem; Hipp, hippocampus; Cb, cerebellum.

To confirm the selectivity of TAK-063 for native PDE10A, *in vitro* ARG with [^3^H]TAK-063 was performed using rat brain sagittal sections. The chemical structure of [^3^H]TAK-063 is shown in Fig. 1A. For anatomical identification, HE staining was conducted using adjacent sections (Fig. 1B). The radioactivity of [^3^H]TAK-063 was selectively detected in the CPu, NAc, the globus pallidus (GP), and the substantia nigra (SN), where PDE10A is highly expressed. Radioactivity was also detected in the connecting pathway between the striatal complex and SN (Fig. 1C). Following this, we investigated the inhibition of [^3^H]TAK-063 accumulation by using PDE10A inhibitors with different chemical structures: MP-10 and non-radiolabeled TAK-063. The selective accumulation of [^3^H]TAK-063 at 8 nM was mostly blocked by 1 μM of either MP-10 or TAK-063 (Fig. 1D and E). The amount of [^3^H]TAK-063 radioactivity in several brain regions in the absence or presence of these cold compounds was represented as PSL value (/mm^2^) (Fig. 1F). In the presence of 1 μM of MP-10, [^3^H]TAK-063 radioactivity was significantly decreased in the CPu (*P* ≤ 0.01), NAc (*P* ≤ 0.01), and Hipp (*P* ≤ 0.05). The PSL values in the presence of 1 μM of MP-10 were considered as backgrounds and the specific binding of [^3^H]TAK-063 in the CPu, NAc, and Hipp was calculated using these PSL values. High specific binding was observed in the CPu and NAc with PSL values of 175 ± 21.3 and 88.2 ± 20.1, respectively. The PSL value in the Hipp was only 4.26 ± 0.784, which was more than 40-fold lower than that in the CPu. These results suggest that [^3^H]TAK-063 selectively binds to native PDE10A in rat brain sections.

### *In Vitro* ARG with [^3^H]TAK-063 in Mouse Brain Sections

**Fig 2:**
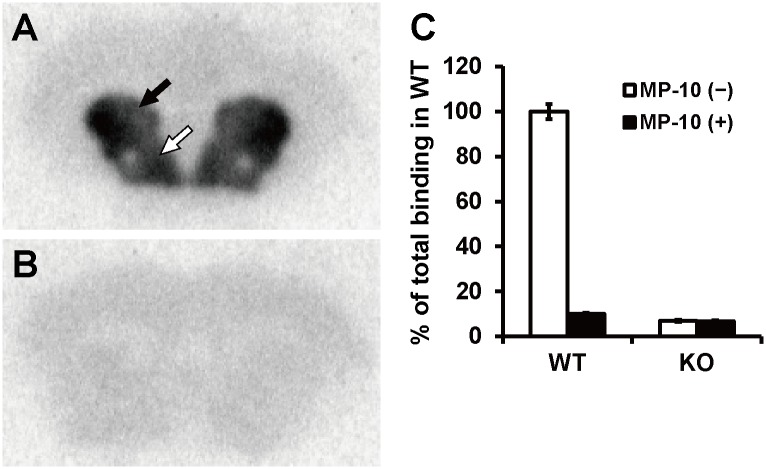
*In vitro* autoradiography (ARG) using [^3^H]TAK-063 in mouse brain sections. [^3^H]TAK-063 selectively accumulated in the caudate putamen (CPu; black arrow) and nucleus accumbens (NAc; white arrow) of wild-type (WT) mouse brain sections (A). The selective accumulation of [^3^H]TAK-063 in these areas did not occur in *Pde10a*-KO mouse brain sections (B). Radioactivity levels in the CPu of brain sections in the presence and absence of an excess amount of MP-10 are represented as a percent of total binding of WT mice (C). Data are represented as mean ± SEM.

We next performed *in vitro* ARG using [^3^H]TAK-063 and coronal brain sections from *Pde10a* WT and KO mice. In WT mouse brain sections, [^3^H]TAK-063 selectively accumulated in the CPu and NAc, where PDE10A is highly expressed (Fig. 2A). This selective accumulation was not observed in brain sections from *Pde10a* KO mice (Fig. 2B). We also conducted a blocking experiment with an excess amount of MP-10 (1 μM) using brain slices from these mice. In the presence of 1 μM of MP-10, [^3^H]TAK-063 radioactivity in the CPu of WT mouse brain sections was similar to that in KO mouse brain sections (Fig. 2C). These results further demonstrate the specific binding of [^3^H]TAK-063 to native PDE10A.

### Binding Affinity of [^3^H]TAK-063 for Native PDE10A in Rat Brain Sections

**Fig 3:**
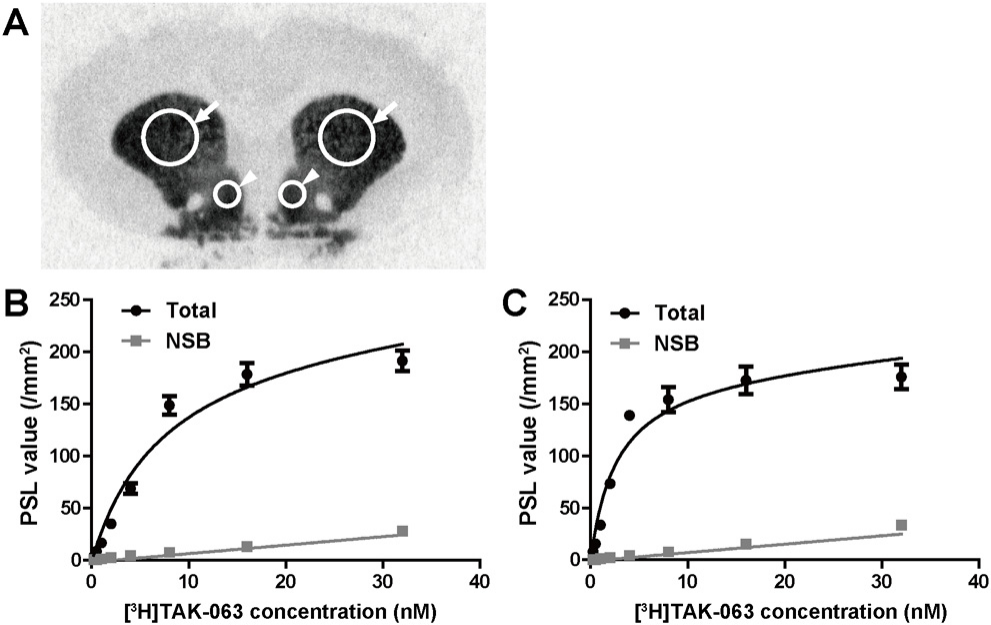
Saturation binding analysis using [^3^H]TAK-063 in rat brain coronal sections. A saturation binding assay was performed with a range of concentrations of [^3^H]TAK-063. Regions of interest (ROIs) were the bilateral caudate putamen (CPu; arrows) and nucleus accumbens (NAc) shell (arrowheads) in the autoradiograms (A). Total and non-specific binding in each ROI was represented as PSL values (/mm^2^), and saturation binding curves from the CPu (B) and NAc shell (C) were analyzed by nonlinear regression. K_d_ values in the CPu and NAc shell were estimated at 7.2 ± 1.2 nM and 2.6 ± 0.5 nM, respectively. All data were represented as mean ± SEM.

We next evaluated the binding affinity of [^3^H]TAK-063 to native PDE10A. We conducted a saturation binding assay using rat brain coronal sections and calculated K_d_ values in the CPu and the shell region of NAc. ROIs in a rat brain section were shown in Fig. 3A. Selective and saturable binding of [^3^H]TAK-063 was observed in these regions with mean ± SEM K_d_ values of 7.2 ± 1.2 nM for the CPu and 2.6 ± 0.5 nM for the NAc shell (Fig. 3B and C), suggesting that [^3^H]TAK-063 binds to a single high-affinity site of PDE10A in the rat brain.

### *In Vivo* ARG with [^14^C]TAK-063 in Rats

**Fig 4:**
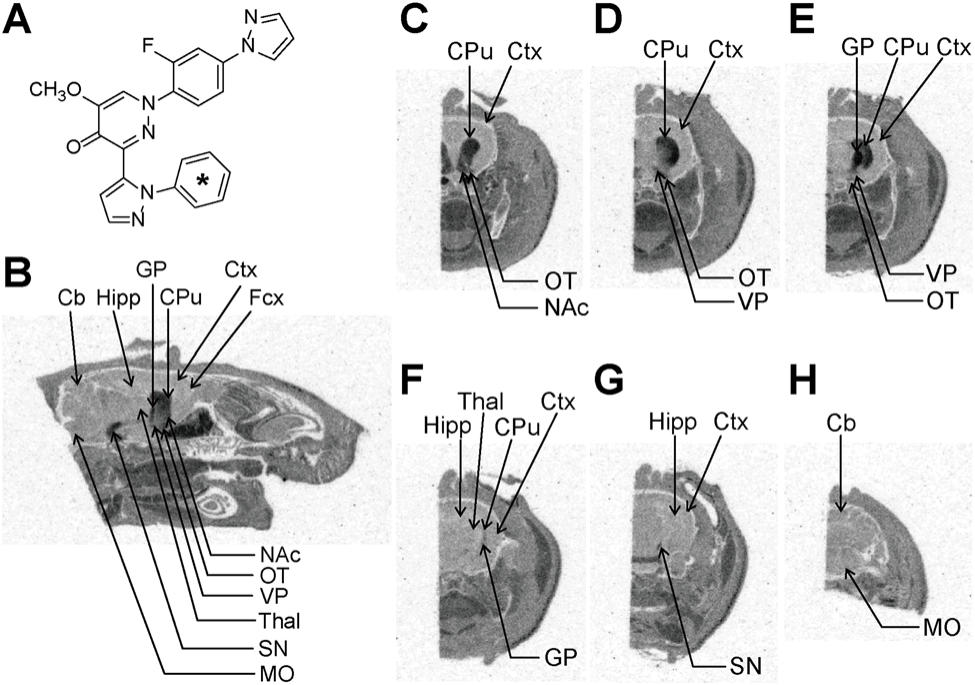
*In vivo* ARG of [^14^C]TAK-063 in rats. The chemical structure of [^14^C]TAK-063 (A). The asterisk denotes the labeled position. Autoradiograms of head sections were obtained from male rats 6 h after single oral administration of [^14^C]TAK-063. The autoradiograms of 40 μm sagittal sections between 2.1 to 2.4 mm lateral to midline were taken (B). The locations for each coronal section relative to the bregma were 1.7 to 1.2 mm (C), 0.48 to −0.26 mm (D), −0.4 to −0.8 mm (E), −2.8 to −3.1 mm (F), −6.0 to −6.3 mm (G), and −12.7 to −12.8 mm (H). Acc, nucleus accumbens; Cb, cerebellum; Cpu, caudate putamen; Ctx, cortex; Fcx, frontal cortex; GP, globus pallidus; Hipp, hippocampus; MO, medulla oblongata; OT, olfactory tubercle; SN, substantia nigra; Thal, thalamus; VP, ventral pallidum.

To validate PDE10A selectivity of TAK-063 *in vivo*, *in vivo* ARG was conducted after oral administration of [^14^C]TAK-063. The chemical structure of [^14^C]TAK-063 is shown in Fig. 4A. Six hours after oral administration of [^14^C]TAK-063 (1.5 mg/kg), autoradiograms of sagittal and coronal head sections were obtained. High radioactivity was observed in the CPu, NAc, GP, and SN of the rat brain regions (Fig. 4B-H), consistent with those in which [^3^H]TAK-063 accumulated in *in vitro* ARG studies and with areas of PDE10A high expression in the rat brain. Thus, [^14^C]TAK-063 appears to selectively bind to native PDE10A protein *in vivo*.

### *In Vivo* Occupancy Study of TAK-063 in Rats

**Fig 5:**
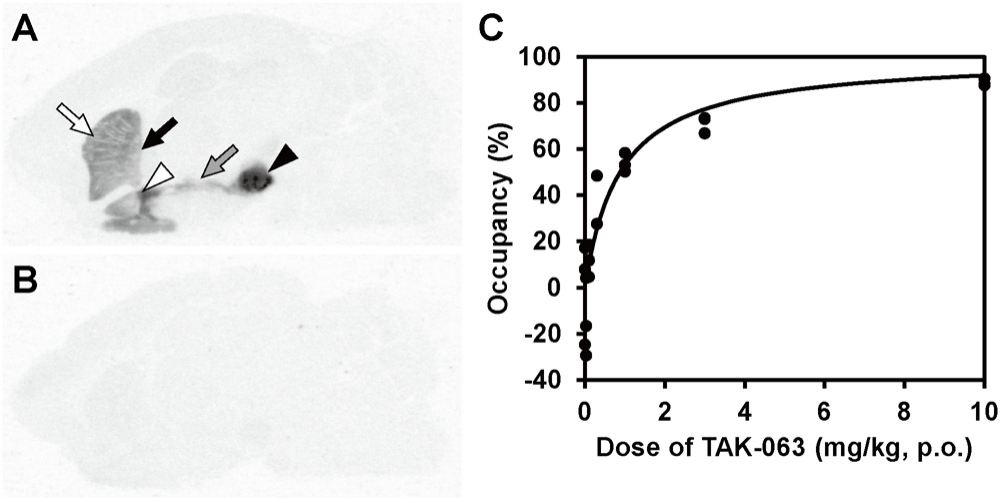
*In vivo* occupancy study of TAK-063 using T-773 as a tracer in rats. In *in vitro* ARG study using the rat brain sagittal section, [^3^H]T-773 selectively accumulated in the caudate putamen (CPu; white arrow), globus pallidus (GP; black arrow), nucleus accumbens (NAc; white arrow head), substantia nigra (SN; black arrow head), and striatonigral projection (gray arrow), where PDE10A is highly expressed (A). This accumulation was almost completely blocked by an excess amount of TAK-063 (B). PDE10A occupancy (%) in the striatum was plotted against doses of orally administered TAK-063 in rats with intravenous T-773 injection (C). The cerebellum was used as a reference region. PDE10A occupancy was increased in a dose-dependent manner. Oral administration of 0.88 mg/kg of TAK-063 resulted in 50% PDE10A occupancy as determined by regression analysis.

PDE10A occupancy by TAK-063 was measured using T-773 as a tracer. First, we investigated whether TAK-063 could compete with PDE10A-selective binding of T-773. [^3^H]T-773 selectively accumulated in PDE10A-expressing regions in sagittal brain sections (Fig. 5A), and the accumulation was almost completely blocked in the presence of an excess amount of TAK-063 (Fig. 5B), indicating binding competition between [^3^H]T-773 and TAK-063.

*In vivo* occupancy study using non-radiolabeled tracer is an established method which confers several advantages over those using radiolabeled tracers in living animals, such as no risk of contamination by radionuclides and no influence of radiometabolites [31,32]. Therefore, PDE10A occupancy by TAK-063 was measured using non-radiolabeled T-773 as a tracer. Specific binding of T-773 to PDE10A in the Str was calculated using the Cb as a reference region based on the lack of specific binding of [^3^H]TAK-063 (Fig. 1); amount of T-773 that specifically bound to PDE10A in the Str was determined by taking the difference in its concentration between the Str and Cb. Striatal PDE10A occupancy by TAK-063 was calculated from the reduction in the binding amount of T-773 to PDE10A after administration of various dosage of TAK-063. The Tmax of orally administered TAK-063 in the rat brain was 2 h in our preliminary study. Therefore, non-radiolabeled T-773 was intravenously injected to rats 90 min after oral administration of TAK-063, and then the Str and Cb were collected from each rat 30 min after T-773 injection. The striatal concentration of T-773 without TAK-063 administration was more than 5-fold higher than that of the reference area, Cb, suggesting the Str-selective accumulation of T-773 (Table 3). The concentration of T-773 in the Str was dose-dependently decreased by pretreatment with TAK-063 (Table 3). When the Cb was employed as a reference site, the calculated PDE10A occupancy by TAK-063 in the Str was increased in a dose-dependent manner (Fig. 5C). When data were fitted by nonlinear regression, PDE10A occupancies at 0.3 and 3 mg/kg of TAK-063 were 26% and 77%, respectively. TAK-063 (0.88 mg/kg) was estimated to produce 50% PDE10A occupancy within the Str.

**Table 3:** Concentration of T-773 in the rat brain and displacement by TAK-063.

	Dose of TAK-063 (mg/kg, p.o.)						
**Brain area**	**0**	**0.03**	**0.1**	**0.3**	**1**	**3**	**10**
**Striatum**	26.1 ± 2.6	28.5 ± 2.0	22.2 ± 0.9	15.9	12.9 ± 0.9	8.9 ± 0.4	5.3 ± 0.4
**Cerebellum**	5.0 ± 0.4	4.4 ± 0.4	3.6 ± 0.2	2.8	3.2 ± 0.4	2.8 ± 0.1	2.9 ± 0.6

The data (ng/g tissue) are represented as mean (n = 2 at 0.3 mg/kg) or mean ± SEM (n = 3).

## Discussion

TAK-063 showed potent inhibitory activity (IC_50_ value of 0.30 nM) and high selectivity (more than 15000-fold against other PDEs) for human recombinant PDE10A2 *in vitro* [24]. Moreover, TAK-063 did not induce a significant response when tested for activity against 91 enzymes and receptors except for PDE family even at 10 μM, which is more than a 33000-fold higher concentration than the IC_50_ value for recombinant PDE10A2 (0.30 nM). Thus, TAK-063 is highly selective for recombinant PDE10A *in vitro*.

We performed ARG using radiolabeled TAK-063 and rodent brain sections to confirm the selectivity of TAK-063 for native PDE10A. PDE10A is highly enriched in striatal MSNs [10]. MSNs constitute the direct and indirect pathways projecting to the SN and GP, respectively [30]. High levels of accumulation of [^3^H]TAK-063 were observed in the CPu, NAc, GP, SN, and the striatonigral projection of the rat brain, supporting the selective accumulation of [^3^H]TAK-063 in striatal MSNs. MP-10 was previously reported to interact with PDE10A at the substrate-binding site in the catalytic domain [27]. Co-crystal structural analysis of TAK-063 with the PDE10A catalytic domain showed that TAK-063 also uses this binding site [24]. Therefore, we performed a blocking study using MP-10 as a control to confirm PDE10A-selective binding of [^3^H]TAK-063 in an *in vitro* ARG study. As expected, [^3^H]TAK-063 accumulation in rat brain sections was almost entirely blocked by an excess amount of either non-radiolabeled TAK-063 or MP-10. Furthermore, the Str-selective accumulation of [^3^H]TAK-063 was almost completely abolished in brain sections from *Pde10a* KO mice with complete deletion of PDE10A protein [25]. These results indicate the PDE10A-specific binding of TAK-063 under physiological conditions.

In previous immunohistochemical studies with rat brain sections, PDE10A immunoreactivity was detected in parts of the Hipp, Cb, and cortex, as well as the CPu, NAc, GP, and SN [4,5]. In those reports, PDE10A expression was confined to individual neuronal nuclei in the Hipp, Cb, and cortex. PDE10A expression levels were 50–200-fold lower in the Hipp, Cb, and cortex than that in the Str [4,5]. In the present autoradiography study, specific binding of [^3^H]TAK-063 in the Hipp was observed at more than 40-fold lower levels than that in the CPu, and no specific binding was observed in the Fcx, Thal, Bs, and Cb. Thus, PDE10A may be expressed in the Hipp at more than 40-fold lower levels than that in the CPu. PDE10A expression levels in the other non-striatal regions are under detection limit at the level of sensitivity of the present study.

A saturation binding assay using rat brain sections showed that [^3^H]TAK-063 bound to a single high-affinity binding site with K_d_ values of 7.2 and 2.6 nM in the CPu and NAc shell, respectively. It is reasonable to determine NSB by the addition of 1 μM of MP-10 because this concentration of MP-10 almost completely inhibited the selective binding of [^3^H]TAK-063 in the blocking study. Indeed, [^3^H]TAK-063 binding in the presence of 1 μM of MP-10 was linear in both the CPu and NAc shell over the range of concentrations used, suggesting NSB. As discussed before, the TAK-063-binding site in the PDE10A enzyme is the substrate-binding site in the catalytic domain; thus, the high binding affinity of TAK-063 suggests potent inhibitory activity against native PDE10A.

Orally administered [^14^C]TAK-063 selectively accumulated in rat brain areas associated with high PDE10A expression in the *in vivo* ARG study. This result suggests that systemically administered TAK-063 can penetrate the blood-brain barrier and specifically bind to native PDE10A in living rats. We measured PDE10A occupancy by TAK-063 using T-773, a brain penetrable PDE10A-specific tracer, with the Cb as a reference region [25]. The *in vitro* competitive binding study revealed that PDE10A-selective accumulation of [^3^H]T-773 can be inhibited by TAK-063; thus, PDE10A occupancy by TAK-063 can be measured using T-773 displacement. Fitted by nonlinear regression, a dose of 0.88 mg/kg of TAK-063 was estimated to produce 50% PDE10A occupancy within the striatum.

In summary, we demonstrated that TAK-063 is specific for both recombinant PDE10A *in vitro* and for native PDE10A *in vivo*. Furthermore, PDE10A occupancy of orally administered TAK-063 was successfully calculated using T-773 as a tracer in rats. Pre-clinical and clinical investigations of the therapeutic potential of TAK-063 against CNS disorders such as schizophrenia and HD with accurate information regarding PDE10A occupancy will improve our understanding of the relation between enzyme occupancy and the pharmacodynamic effects of PDE10A inhibitors and provide important information regarding this translational approach. TAK-063 is currently being clinically evaluated for the treatment of schizophrenia.
